# Associations between atopic dermatitis and other disorders

**DOI:** 10.12688/f1000research.12975.1

**Published:** 2018-03-12

**Authors:** Jonathan I. Silverberg

**Affiliations:** 1 Departments of Dermatology, Preventive Medicine, and Medical Social Sciences, Northwestern University Feinberg School of Medicine, Arkes building, 676 N Saint Clair St, Suite 1600, Chicago, IL, 60611, USA

**Keywords:** atopic dermatitis, comorbidities, eczema

## Abstract

Atopic dermatitis is a heterogeneous, chronic, and inflammatory skin disease that is associated with a profound symptom burden. Recent studies have demonstrated associations between atopic dermatitis and a number of multi-organ and systemic disorders. The origin of these comorbid conditions is likely multifactorial, with combined effects of skin-barrier disruption, immune dysregulation, intense symptoms, and iatrogenic complications. Some of these comorbid conditions appear to be related to the underlying severity of atopic dermatitis and inadequate disease control. This review will summarize recent developments in the understanding of the comorbid health disorders associated with atopic dermatitis, particularly infections, sleep disturbances, and cardiovascular disease.

## Introduction

Atopic dermatitis (AD) is a chronic inflammatory skin disease that is associated with a heterogeneous and often variable constellation of symptoms and signs. The symptoms of AD include cutaneous itch and pain ^1^, sleep disturbance and fatigue ^2, 3^, and mental health symptoms ^4– 6^. The cutaneous signs of AD include erythema (redness), lichenification (accentuation of the skin lines from chronic rubbing), scaling, oozing or weeping, and prurigo (pickers) nodules. Together, these contribute toward profound functional disturbances that limit the ability to perform activities of daily living and cause psychosocial distress and stigma.

However, AD appears to have effects that are not just “skin deep”. Many patients have comorbid infectious ^7, 8^, autoimmune ^9^, respiratory, neuropsychiatric ^10– 12^, musculoskeletal ^13, 14^, and potentially even cardiovascular disorders ^15– 19^. Some authors have wondered whether AD is a systemic disease, i.e. with global impact beyond the immediate skin signs and symptoms ^20^. This review will summarize recent developments in the understanding of the comorbid health disorders associated with AD.

## Methods

Reviewed articles were identified by performing a PubMed search for “comorbid*” and (“atopic dermatitis” or eczema). Original research and review articles were selected for this non-systematic review.

## Infections

There are a number of potential risk factors for infections to occur in AD patients, including skin-barrier dysfunction secondary to decreased expression of epidermal lipids and mechanical disruption from scratching ^21^, decreased expression of anti-microbial peptides ^22^, aberrant Toll-like receptor signaling and innate immunity ^23, 24^, increased colonization with *Staphylococcus aureus* in lesional and non-lesional skin ^25, 26^, and use of topical and/or systemic immunosuppressing medications (e.g. corticosteroids, calcineurin inhibitors, etc.). Indeed, there is a well-recognized association between AD and skin infections, e.g. molluscum contagiosum and eczema herpeticum ^27– 29^; this is one of the minor Hanifin & Rajka diagnostic criteria ^30^.

Recent studies have demonstrated that AD is also associated with extra-cutaneous infections ^7, 8, 31, 32^. First, a study of 91,642 children from the 2007–2008 National Survey of Children’s Health (NSCH) found that children with AD had higher rates of recurrent ear infections ^32^. Subsequently, a study of 9,417 children from the 2007 National Health Interview Survey (NHIS) found that children with AD (with or without atopic disease) had significantly higher odds of influenza/pneumonia, sinus infections, head or chest colds, and strep throat ^7^, whereas only children with AD and other atopic disease had higher odds of warts ^7^. Next, a study of 34,613 adults from the 2012 NHIS found that adults with AD had significantly higher odds of influenza/pneumonia, strep throat, head or chest cold, sinus infection, gastroenteritis, and chickenpox ^8^. These population-based studies demonstrated consistently increased risk of extra-cutaneous infections. However, they employed surveys, which may be vulnerable to misclassification bias. That is, it is possible that some subjects with self- and/or caregiver-reported AD actually had a different dermatosis. However, previous studies observed good concordance between self-reported AD and physician-diagnosed AD ^33, 34^. Furthermore, we carried out a multicenter study in order to validate the question used in the NSCH and established that the question had a sensitivity and specificity of 0.70 and 0.96, respectively, for AD ^35^. It is significant that the question has a high specificity, signifying that those who responded positively were very likely to have AD. Thus, we believe that self- and/or caregiver-reported AD is sufficiently valid for an epidemiologic study. Nevertheless, there might be some concern for the misclassification of AD patients who respond negatively to the respective questions.

Most recently, a study of the 2002–2012 National Inpatient Study, including a representative hospital cohort in the US (n=72,108,077 adult discharges), found that AD was significantly associated with 32 of 38 serious infections examined, including cutaneous, upper respiratory, lower respiratory tract or lung, cardiac, brain, bone, and gastrointestinal infections ^31^. The cutaneous infections with the largest effect sizes were eczema herpeticum, erysipelas, and cellulitis. Extra-cutaneous, multi-organ, and systemic infections included encephalitis, endocarditis, infectious arthropathy, enterocolitis, and septicemia. AD was associated with even higher odds of most infections other than psoriasis, including eczema herpeticum, erysipelas, cellulitis, herpes simplex and zoster, acute pharyngitis, bronchitis, endocarditis, and septicemia.

Of note, comorbidities may be more likely found in hospital datasets owing to detection bias (i.e. people are more likely to receive further diagnoses once they are first diagnosed in the medical system) and inclusion of patients with more severe disease. However, these potential biases are unlikely in population-based studies. Taking the population-based and confirmatory hospital-based studies together, there appear to be higher rates of extra-cutaneous and systemic infections in both children and adults with AD.

Several important questions remain to be answered. First, what are the precise mechanisms of increased infections in AD, particularly extra-cutaneous infections? Can such infections be prevented with improved long-term control of the signs and symptoms of AD? Are such infections largely iatrogenic? Are there additional strategies that are effective at preventing serious infections in AD? Future basic science, translational, and clinical research are currently underway that will hopefully shed light on these important matters.

## Sleep disturbance

There are a number of potential risk factors for sleep disturbance in AD patients, including the itch-scratch cycle ^36^, poor sleep hygiene, circadian rhythm-induced modification of itch ^37^, and secondary effects of inflammatory cytokines on sleep regulation ^38^.

Earlier studies have shown substantially impaired quality of sleep in both childhood ^39– 46^ and adult ^33– 35, 47, 48^ AD, with reduced sleep overall, more regular and protracted awakening, overall decreased sleep efficiency, and increased daytime dysfunction being reported. Actigraphy and infrared video (objective measures of sleep) revealed fragmented sleep with more night-time awakenings, prolonged awakenings, lower overall sleep efficiency, increased scratch time, and restless nocturnal movement in both adults and children with AD ^33, 36^.

In childhood AD, sleep disturbance was the second most burdensome symptom after itch ^40^ and, in terms of quality of life (QOL), is one of the most troublesome factors ^49, 50^. The International Study of Life with Atopic Eczema performed a multicenter questionnaire-based study including 1,098 adults and discovered that patients had an average of 8.4 nights of disrupted sleep with a typical AD flare, which worked out as ~81 days per patient-year ^35^. A study of 34,613 adults from the 2012 NHIS found that 25–33% of US adults with self-reported eczema reported fatigue, regular daytime sleepiness, and regular insomnia ^2^. Adults with self-reported eczema were also more likely to report either short or long sleep duration ^2^. The authors found that eczema and fatigue, sleepiness, and insomnia were significant predictors of poorer overall health, number of sick days, and doctor visits, with eczema in combination with sleep symptoms associated with a greater chance of worse outcomes than either sleep symptoms or eczema alone ^2^. A study of 5,563 adults from the National Health and Nutrition Examination Survey (NHANES) found that US adults with AD more commonly reported short sleep duration, trouble falling asleep, night-time awakenings, early morning awakenings, and leg jerks and leg cramps during sleep and were more likely to feel unrested, being overly sleepy during the day and feeling as if they did not get enough sleep ^3^. Sleep disturbances may have very detrimental effects in AD patients, including poor performance at school and work, impaired health-related QOL (HRQOL) ^33, 40, 49– 51^, considerable economic burden ^33, 49– 52^, and increased risk of psychological disorders ^53, 54^, motor vehicle accidents, and workplace injury ^55– 57^. Together, these studies suggest that sleep disturbances are both very common and burdensome in AD patients and warrant interventions for their treatment and prevention.

In addition, as elaborated below, sleep disturbances may be a major driver for developing other comorbid health conditions, including cardiovascular risk and events.

## Cardiovascular risk factors and events

There are multiple potential risk factors for excess cardiovascular and cerebrovascular risk in AD patients, including chronic sleep disturbance ^2, 3^, sedentary physical activity ^58, 59^, higher rates of cigarette smoking ^60^ and alcohol consumption ^15^, and adverse effects from some systemic treatments (e.g. corticosteroids and cyclosporine A).

Multiple studies found associations between AD and obesity in children and adults from clinical and US population-based cohorts ^18, 19, 61, 62^. Given that there was an abundance of published studies on the topic, we performed a systematic review and meta-analysis to determine if there was a consistent association between AD and obesity; 30 studies were identified ^63^. We found that patients who were overweight and/or obese had higher odds of AD than normal-weight patients. These results were significant in both children and adults and in studies conducted in North America and Asia but not Europe ^63^. These results provide the highest level of evidence to support an association among AD, being overweight, and obesity.

Sleep disturbance in-and-of-itself has been linked to increased risk of cardiovascular disease and/or mortality ^64, 65^. A meta-analysis of 13 prospective studies including 122,501 subjects found that difficulty in initiating or maintaining sleep or presence of restless, disturbed nights of sleep were associated with a 45% increased risk of developing or dying from cardiovascular disease ^64^. Another meta-analysis of 141 reports including 3,582,016 subjects found that both short (<7 hours per night) and long sleep duration (≥9 hours per night) were associated with increased all-cause mortality and cardiovascular events ^65^. It is logical that sleep disturbances in AD would play an important role in the development of excess cardiovascular risk.

In one of the seminal studies on the association of AD and cardiovascular risk, we analyzed data from 27,157 and 34,525 adults aged 18–85 years from the 2010 and 2012 NHIS, respectively. We found that adults with self-reported eczema had higher odds and earlier age of initiation of cigarette smoking, higher odds of consumption of alcoholic beverages, lower odds of daily vigorous physical activity and lower frequency of vigorous physical activity in the past week, and higher prevalence of class II/III obesity, hypertension, prediabetes, diabetes, and high cholesterol ^15^. Moreover, there was considerable correlation between self-reported eczema and sleep disturbances, with eczema linked to fatigue, daytime sleepiness, or insomnia being connected with an even greater likelihood of obesity, hypertension, prediabetes, diabetes, and elevated cholesterol levels than eczema alone. These results were very provocative and reproducible across both cohorts, but AD and cardiovascular risk factors were self-reported, potentially leading to misclassification.

We then sought to determine the association between AD and high blood pressure in children. We performed a multicenter, pediatric dermatology practice-based case-control study of 132 US children and adolescents with active moderate-to-severe AD and 143 healthy controls ^16^. We found that moderate-to-severe AD was associated with central obesity, as judged by elevated body mass index (BMI), waist circumference ≥85th percentile, and waist-to-height ratio of ≥0.5. AD was also associated with higher systolic and diastolic blood pressure for age, sex, and height percentiles. AD overall, and particularly severe/very severe AD, were associated with higher odds of a systolic blood pressure ≥90th percentile. An interesting observation was that elevated systolic and diastolic blood pressure were associated with obesity in the healthy controls but not the AD patients. This suggests that blood pressure elevations occurring in AD may be independent of adiposity. It was also interesting that AD was particularly associated with higher systolic blood pressure in Hispanic/Latino and Asian patients, suggesting that there may be racial/ethnic differences in these associations. This is an important consideration when interpreting studies from cohorts with an underrepresentation of these racial/ethnic groups.

We then sought to determine whether the association between AD and cigarette smoking was consistent across the scientific literature. To do so, we performed a systematic review and meta-analysis (n=86 observational studies) and found that AD was associated with higher odds of active smoking and exposure to passive smoke but not maternal smoking during pregnancy ^60^. Together, these results provide the highest level of evidence that AD is associated with both active and passive exposure to cigarette smoke.

Next, we sought to confirm the association between AD and decreased physical activity. First, we performed a systematic review of the literature to determine whether AD was consistently associated with decreased physical activity ^66^. Unfortunately, we found that few studies were performed, with variable study designs, methodological rigor, and results. It became clear that more studies were needed in this area.

Thus, we studied the association between childhood AD (as well as asthma and hay fever) and physical activity. We analyzed data from two cross-sectional studies including 133,107 children aged 6–17 years enrolled in the 2003–2004 and 2007–2008 NSCH and found that caregiver-reported eczema, particularly severe eczema, was associated with decreased odds of vigorous physical activity ^59^. Moderate and severe eczema and eczema associated with sleep disturbance (only 0–3 nights of adequate sleep) were associated with decreased odds of sports participation in the past year. Severe eczema and eczema associated with sleep disturbance (only 0–3 nights of adequate sleep) were also associated with increased odds of having 5 or more hours of daily screen time (television and video games) ^59^. One of the limitations of this study was that physical activity was assessed using caregiver reports, which may be imprecise. Our next study analyzed data from 3,252 adults aged 18–85 in the 2005–2006 NHANES whose daily physical activity was objectively measured using actigraphy (or accelerometry). We found that AD was associated with significantly lower average total counts of daily activity and less moderate-to-vigorous physical activity but not associated with sedentary time or light physical activity ^58^. Together, these data indicate that AD is associated with decreased vigorous physical activity and/or increased sedentary activity in both US children and adults.

Finally, given the observed increase in cardiovascular risk factors, we sought to determine whether AD was associated with increased occurrence of cardiovascular disease and events *per se*. We analyzed data from three US population-based cohorts: the 2005–2006 NHANES (n=4,970) and the 2010 (n=27,157) and 2012 (n=34,525) NHIS. In NHANES, flexural eczema in the past year (using the International Study of Asthma and Allergies in Childhood definition of AD) was associated with significantly higher odds of coronary artery disease, heart attack, and congestive heart failure, but not with stroke, whereas in NHIS 2010 and 2012, self-reported 1-year history of eczema was associated with significantly higher odds of coronary artery disease, angina, heart attack, other heart disease, stroke, and peripheral vascular disease ^17^. These associations remained significant in multivariable models that controlled for asthma and hay fever or BMI, history of ever smoking cigarettes, consumption of alcohol in the past year, and vigorous activity in the past 30 days as covariates. While these results were reproducible across three cohorts, AD and cardiovascular risk factors were self-reported, potentially leading to misclassification.

A subsequent study of adults with AD (n=31), psoriasis (n=58), and retrospectively matched controls (n=33) found increased coronary artery calcium scores using cardiac computed tomography angiography (CCTA) 18-segment models of the coronary tree and mild single-vessel disease in adults with AD compared with both normal and psoriasis patients ^67^. However, patients with psoriasis had more coronary stenosis and 3-vessel or left main artery disease compared to those with AD. These results demonstrate increased cardiovascular risk using objective assessments.

Subsequently, AD was reported to be an independent risk factor for ischemic stroke in a large Taiwanese population-based study ^68^. Similarly, using the Danish national patient registry, patients with severe AD were found to have increased risk of cardiovascular disease ^69^. However, the association did not remain significant in multivariable models controlling for socioeconomic status, smoking, comorbidities, and medication use, suggesting that the observed differences in cardiovascular risk are related to lifestyle factors rather than AD-associated inflammation. In a recent study of US female nurses, non-fatal stroke was significantly associated with *ever* history of AD in models that controlled for age ^70^. However, stroke was no longer significant after controlling for hypertension, hypercholesterolemia, and diabetes. This suggests that these cardiovascular risk factors are likely responsible for the increased stroke in AD and may be part of the causal model. In contrast, AD was not associated with non-fatal myocardial infarction. However, this study had several major limitations, including lack of assessment of current versus past history of AD, lack of assessment of AD severity, and lack of generalizability to the US population, as it was based on a cohort of female nurses. Two important points are needed to properly interpret these studies. First, AD appears to indirectly increase the risk of cardiovascular disease and events through multiple cardiovascular risk factors. That is, AD is associated with increased odds or risk of obesity, hypertension, high cholesterol, diabetes, smoking, etc. These are all well-established causes of heart disease and appear to be the causal pathway for association between AD and cardiovascular disease. Thus, the abovementioned study from Denmark ^69^ and the Nurses Health Study ^70^ that show loss of significant association in multivariable models controlling for cardiovascular risk factors require cautious interpretation. Even in those studies, AD patients had higher risk of cardiovascular disease and/or events. However, the risk is mediated by the cardiovascular risk factors. Taken together, most studies support at least an indirect and possibly direct association between AD and increased cardiovascular risk.

Recently, we analyzed data from the 2002–2012 National Inpatient Sample, including a representative 20% sample of all US hospitalizations (n=72,108,077 adults). We found that AD was associated with significantly increased odds of cardiovascular risk and disease, similar to that observed in psoriasis, hidradenitis, pemphigus, and pemphigoid ^71^. In particular, AD was associated with increased odds of hypertension, obesity, congestive heart failure, peripheral vascular disease, peripheral and vascular atherosclerosis, pulmonary circulation disorders, late effects of cerebrovascular disease, and other cerebrovascular disease ^71^. This study confirms an association between physician-diagnosed AD and cardiovascular/cerebrovascular disease. However, given that this was a hospital cohort, it is likely that the included patients had more severe AD.

Together, the results of the above-mentioned studies demonstrate that AD is associated with increased cardiovascular risk. The association is likely multifactorial, with contributions from chronic sleep disturbance, decreased physical activity, and increased cigarette smoking and alcohol consumption, etc. A number of important questions remain despite the growing body of evidence in support of an association of AD and cardiovascular disease. Is the excess cardiovascular risk in AD patients modifiable? That is, can one or more interventions be implemented that might reduce AD patients’ cardiovascular risk? Can optimized control of AD signs and symptoms mitigate the risk of cardiovascular disease in AD? Future studies are needed to answer these questions and potentially improve overall health outcomes of AD patients. This is an exciting and rapidly developing area of research.

## Additional considerations

It is important to note that the effect sizes for associations between AD and cardiovascular and other comorbidities were modest for some outcomes (odds or hazard ratios ≤2). That is, only a small subset of AD patients may actually develop these comorbidities. Moreover, it is important to consider that very large datasets, such as those used in the abovementioned studies, are overpowered to detect significant associations, even with modest effect sizes. Thus, it is imperative that different associations be reproducible across multiple cohorts, study designs, and methods. Of note, most of the abovementioned associations have been reproduced across more than one cohort or study. However, a key question that remains is how to identify or predict those patient subsets with higher risk of various comorbidities.

## Conclusions

AD is associated with many comorbid health conditions, include extra-cutaneous, multi-organ, and systemic infections as well as cardiovascular disease (see Table 1). Together, these suggest that AD is indeed a systemic disease. The origin of these comorbid conditions is likely multifactorial, with combined effects of skin-barrier disruption, immune dysregulation, profound symptom burden, and iatrogenic complications. Many of these comorbid conditions are directly related to the underlying severity of AD and inadequate disease control. Consequently, many of these comorbidities may be clinically relevant and prevented or mitigated by optimizing control of the AD. On the other hand, some of these comorbidities may not be avoidable. Nevertheless, they may still pose significant jeopardy to the health of AD patients, thereby warranting increased surveillance, early detection, and management. Much research is still needed to better hone our understanding of the relationship between AD and its many comorbid health conditions. Finally, it is essential to identify therapeutic and/or other strategies that might prevent or mitigate increased infectious and cardiovascular risk in AD patients.

**Table 1.** Comorbid disorders that have been shown to be associated with atopic dermatitis in multiple studies.

Disorder
Cutaneous infections (including impetigo, cellulitis, and viral and fungal infections)
Extra-cutaneous and systemic infections (including sepsis)
Sleep disturbances (including difficulty falling asleep and staying asleep, poor sleep efficiency, and poor quality of life related to sleep disturbances)
Cardiovascular risk factors (including obesity, hypertension, high cholesterol, type 2 diabetes, smoking, alcohol consumption, and sedentary activity)
Cardiovascular disease and events (including coronary artery disease, angina, heart attack, congestive heart failure, and stroke)

## Abbreviations

AD, atopic dermatitis; BMI, body mass index; NHANES, National Health and Nutrition Examination Survey; NHIS, National Health Interview Survey; NSCH, National Survey of Children’s Health.
